# Dissecting genotype-by-environment interactions and genetic diversity in gamma irradiation-derived bread wheat (*Triticum aestivum* L.) mutants

**DOI:** 10.3389/fpls.2026.1895559

**Published:** 2026-09-23

**Authors:** Satish Kumar, Gautam Vishwakarma, Tamanna Chauhan, Kiran Pal, Disha Kamboj, Ankita Kailas Kurhade, Chandra Nath Mishra, Rakesh Kumar Bairwa, Ajay Verma, Arun Gupta, Bikram Kishore Das, Ratan Tiwari

**Affiliations:** 1 Indian Institute of Wheat and Barley Research (ICAR), Karnal, India; 2 Nuclear Agriculture and Biotechnology Division, Bhabha Atomic Research Centre, Trombay, Mumbai, India

**Keywords:** gamma irradiation, genotype-by-environment interactions, mutation breeding, wheat yield stability, yellow rust resistance

## Abstract

Climate change and escalating biotic pressures, particularly stripe (yellow) rust, present major challenges to sustainable wheat production in India and globally. To enhance genetic diversity and introduce novel variations, this study utilized mutation breeding by subjecting seeds of the popular wheat variety DBW88 to gamma irradiation. Following rigorous selection through successive generations, twenty promising mutant lines alongside their parent and commercial checks (HD2967, HD3086, WH1105) were evaluated over three consecutive crop seasons under multi-environment trials. The genotypes were screened for different agronomic traits and field resistance against yellow rust using Adult Plant Resistance (APR) and Seedling Resistance Tests (SRT). Phenotypic stability and genotype-by-environment interactions (GEI) were analyzed *via* Additive Main Effects and Multiplicative Interaction (AMMI) models, GGE biplots, and superiority index, while molecular diversity was assessed using Kompetitive Allele-Specific PCR (KASP) SNP markers. The results revealed highly significant effects of genotype, environment, and GEI on grain yield. Thus, the use of gamma radiation is effective in improving the desirable traits, including grain yield. The mutant line DTWM-16 exhibited the highest baseline superiority and yield stability across environments, followed closely by check WH1105 and mutant DTWM-15. Under stressful environments, DTWM-08 and DTWM-16 demonstrated exceptional localized adaptation. Disease screening revealed that 34% of the mutants (including DTWM-03 and DTWM-08) achieved complete rust immunity, successfully breaking the high susceptibility of the parental line DBW88. Furthermore, molecular profiling indicated a narrow overall genetic base, conserving elite parental structures; however, mutants DTWM-10 and DTWM-14 emerged as distinct, highly divergent outliers with unique allelic combinations and heterozygosity patches. In conclusion, this study confirms that induced mutagenesis effectively balances yield stability and robust disease resistance. It is suggested that these traits can be improved beyond the performance of corresponding traits in their parent genotypes. Elite mutants like DTWM-08 and DTWM-16 are excellent candidates for regional cultivation, while divergent mutant lines DTWM-10 and DTWM-14 provide valuable genetic resources for future hybridization panels to broaden the wheat gene pool. The newly produced mutants can also be used to explore the genetic mechanisms of complex traits in the future.

## Introduction

With a commercial value larger than all other crops put together, wheat is currently the most frequently produced grain in the world (Curtis, 2019). Wheat contains minerals, vitamins, and lipids; it is also a good source of dietary fiber and micronutrients (Shiferaw et al., 2013; Lafiandra et al., 2014). While wheat continues to be the world’s primary staple crop, its production faces challenges brought on by climate change. India holds the second position with 117.94 million tons of production and contributes 13% share to the global wheat basket (UPAg, 2025). The degree of genetic diversity and the desired traits affect wheat improvement (Kahrizi et al., 2010). All significant food crops can be improved through induced genetic diversity, and crops with limited genetic diversity benefit most from the application of mutagenesis to produce novel variations (Begna, 2021).

Crop enhancement is a crucial strategy for ensuring that consumers continue to benefit from food and plant-based products. The majority of crops, however, have stagnant seed yields. For effective crop development, a wide variety of genetic, biochemical, and metabolic variation must be produced. The current needs for plant breeding to increase food yields sustainably can be met by mutation breeding. It has been effectively used to increase crop plants’ productivity and output. It is commonly known that physical mutagens, like as gamma radiation, can be used to cause variation (Vishwakarma et al., 2017). Gamma rays, which are well-known for having a high penetration depth and a short wavelength, are electromagnetic irradiations that are created when certain elements undergo radioactive disintegration. By altering the morphological, physiological, biochemical, genetic, and cytological characteristics of cells and tissues, it has significant influence on plant growth and development (Kiani et al., 2022). Agronomically significant features like grain yield and increased tolerance for or resistance to abiotic and biotic stressors are developed thanks to these induced mutations (Zakir, 2018).

The increasing prevalence of climatic stresses poses a substantial threat to Indian wheat crop, necessitating focused research on enhancing genetic variation and to develop improved cultivars that can withstand these challenges (Trethowan et al., 2018; Kumar et al., 2019b). Therefore, developing high yielding and disease resistant wheat cultivars is a pressing challenge for breeders. Traditional breeding methods, while effective, are often slow and limited by the complex inheritance of the traits. Induced mutations offer a potent way to add fresh and practical variety to crop plants’ qualitative and quantitative traits. The method can also be used to fix particular adaptation-related flaws. The regulatory limitations placed on genetically modified species do not apply to mutagenesis, making it a crucial tool in agricultural development (Ahloowalia et al., 2004; Stacy et al., 2021).

Wheat production is strongly influenced by environmental variation, including differences in temperature, moisture availability, disease pressure and other seasonal factors. Therefore, the performance of a genotype may vary considerably across environments, resulting in genotype × environment interaction (GEI). Such interaction is particularly important for grain yield, which is a complex quantitative trait influenced by both genetic and environmental factors. Understanding GEI is therefore essential for identifying genotypes with broad adaptation as well as those showing specific adaptation to particular production conditions. Multi-environment evaluation can help breeders distinguish genotypes that combine high yield with stability from those whose performance is dependent on particular environments (Trethowan et al., 2018; Kumar et al., 2019b).

Mutation breeding provides an alternative approach for generating useful genetic variation, particularly when an elite cultivar already possesses desirable agronomic characteristics but lacks one or more important traits. Induced mutagenesis using physical mutagens such as gamma irradiation can generate variation for quantitative and qualitative traits and has been used in wheat improvement, including the development of resistance to yellow (stripe) rust (Ahloowalia et al., 2004; Vishwakarma et al., 2017). An important advantage of mutation breeding is that selected mutants can retain much of the favorable genetic background and agronomic adaptation of the parent while introducing variation for specific traits of interest. This is particularly relevant in wheat, where improving disease resistance without compromising yield potential and adaptation remains an important breeding objective.

Stripe rust is one of the major biotic constraints to wheat production, and its impact on agronomic parameters can vary with environmental conditions and pathogen populations. Consequently, resistance identified in a mutant under one set of conditions does not necessarily indicate identical agronomic performance or adaptation across environments. Similarly, a mutant showing high yield in a favorable environment may not necessarily maintain its performance under less favorable conditions. Thus, evaluation of induced mutants across contrasting environments or seasons is important for determining the consistency of their yield performance and identifying genotypes with either broad or specific adaptation. Although mutation breeding has been widely used to generate useful variation in wheat, comparatively less attention has been given to the simultaneous evaluation of induced mutants for yield performance, stability and stripe rust resistance across successive production environments. Such information is important for determining whether the variation generated through mutagenesis can be translated into useful breeding material.

Several statistical approaches have been developed to interpret GEI and genotype stability in multi-environment trials. Among these, the AMMI and GGE biplot approaches provide complementary ways of examining the interaction pattern and identifying genotypes with high yield, stability or specific adaptation (Gauch, 2006; Yan and Tinker, 2006; Yan et al., 2007; Gupta et al., 2023). In the present study, these approaches were used together with the superiority index to evaluate the consistency of grain yield performance and to identify genotypes showing broad or environment-specific adaptation. The objective was not only to identify high-yielding mutants, but also to determine whether their yield performance remained consistent across successive seasons and whether favorable yield performance could be combined with resistance to yellow rust.

Therefore, the present study was undertaken to evaluate gamma irradiation-derived mutants developed from the elite wheat cultivar DBW88 for grain yield and associated agronomic traits over three consecutive crop seasons, and to assess their response to yellow rust using adult plant and seedling resistance tests. Specifically, the study aimed to (a) determine the extent of genotype, environment and GEI effects on grain yield; (b) identify mutants with high and stable yield performance as well as those showing specific adaptation; (c) identify mutants combining desirable yield performance with yellow rust resistance; and (d) assess the extent of molecular variation among the mutants in relation to their parental background. The study was intended to determine the breeding value of gamma irradiation-derived mutants and identify promising lines for further evaluation and utilization in wheat improvement.

## Materials and methods

### Development on mutant lines of DBW 88

10000 seeds of wheat variety DBW88 were mutagenized gamma-rays at BARC Mumbai during 2018-19. Wheat seeds were irradiated at three dose (250, 300 and 350 Gy) of Co_60_ radioisotope gamma rays using an GC5000 (BRIT make) irradiator. Doses were selected based on previous radiosensitivity studies giving optimal Growth Reduction responses (reference). M_0_ seeds were raised at Dalan Maidan (HP), during off-Season 2019. Single spikes from each M_1_ plant were harvested, and during main season, 2019-20, approximately 5000 M_2_ plants were raised as spike to row, at Karnal. Selections were made and 1255 lines were selected based on plant type and yellow rust resistance reactions. M_3_ generation was raised at Dalan Maidan during 2020 and agronomically superior lines with yellow rust resistance were selected. Promising 72 lines identified for further evaluation at Karnal during 2020–21 crop season. Out of these mutant lines, 20 best lines were used for further yield and resistance evaluation in subsequent years, i.e., 2021-22, 2022-23 & 2023–24 at Karnal.

### Evaluation of the mutants for yield and component traits

The selected 20 mutants along with parent and commercial checks, HD2967, HD3086 and WH1105, were evaluated for three years at the research farm of ICAR-IIWBR, Karnal (Details of the mutants and the checks is given in **Table 1**). The field experiment was conducted using a randomized complete block design. The plant material was replicated twice, with a plot size of 6rows of 6 meter each and the row-to-row distance of 23cm. The recommended package of practices was followed to raise the healthy crop as defined previously (reference).

**Table 1:** Details of the mutants and the check varieties.

Code	Genotype name	Details/Pedigree
G1	DTWM-01	Direct mutant of DBW88
G2	DTWM-02	Direct mutant of DBW88
G3	DTWM-03	Direct mutant of DBW88
G4	DTWM-04	Direct mutant of DBW88
G5	DTWM-05	Direct mutant of DBW88
G6	DTWM-06	Direct mutant of DBW88
G7	DTWM-07	Direct mutant of DBW88
G8	DTWM-08	Direct mutant of DBW88
G9	DTWM-09	Direct mutant of DBW88
G10	DTWM-10	Direct mutant of DBW88
G11	DTWM-11	Direct mutant of DBW88
G12	DTWM-12	Direct mutant of DBW88
G13	DTWM-13	Direct mutant of DBW88
G14	DTWM-14	Direct mutant of DBW88
G15	DTWM-15	Direct mutant of DBW88
G16	DTWM-16	Direct mutant of DBW88
G17	DTWM-17	Direct mutant of DBW88
G18	DTWM-18	Direct mutant of DBW88
G19	DTWM-19	Direct mutant of DBW88
G20	DTWM-20	Direct mutant of DBW88
G21	WH1105 (C)	Commercial check variety
G22	DBW88 (C)	Parental check variety
G23	HD3086 (C)	Commercial check variety
G24	HD2967 (C)	Commercial check variety

The data were recorded for seven quantitative traits namely plant height (cm), number of tillers per plant, days to heading, spike length (cm), number of spikelets per spike, 1000-grain weight (g) and grain yield (Kg). Observations were recorded from representative plants/spikes selected randomly from each plot, avoiding border plants. The plant height was measured at the time of maturity from ground level up to terminal spikelet excluding the awns. Days to heading calculated as days taken from sowing to emergence of 75% spikes in a plot. The spike length was measured from the bottom to the spike tip without awns. For 1000- grain weight seeds were counted using grain counter and their weight is recorded using electronic balance. Grain yield of each line recorded using weighing balance (VWR^®^ A-Series balances).

### Screening of the mutants for yellow rust resistance

For screening against yellow rust, infector/spreader rows were planted on all the sides of the sowing block and also in between the lines for proper spread of the disease. Epiphytotic for yellow rust were created by injecting the inoculum of the fungus in to the infector plants. For secondary spread of the disease, inoculum was sprayed on the trial during the months of January and February at weekly intervals. *Adult Plant Resistance (APR)* was evaluated based on host response and key epidemiological parameter like coefficient of infection. Disease severity and infection type was recorded three times at 14 days interval from February to March. Assessments were made using modified Cobb’s scale (Peterson et al., 1948). The response of each genotype was classified according to adult plant infection types as Susceptible (S), Moderately Susceptible, Moderately Resistant (MR), Resistant (R), or Immune (0), following the below scale:

### Seedling resistance test

The mutant genotypes along with parent and checks were evaluated against stripe rust pathotypes (110S119, 238S119, 110S84) and leaf rust pathotypes (77-5, 77-9, 104-2, 12-5) at the seedling stage. The seedling resistance test was conducted at ICAR-IIWBR Regional Station Shimla. The observations for rust resistance at seedling stage were recorded after 14 days of inoculation using 0–9 disease rating scale (McNeal, 1971). wherein (i) 0 = immune = no visible symptoms, (ii) 0; = nearly immune = hypersensitive necrotic or chlorotic fleck present (iii) 1 = highly resistant = small uredinia surrounded by necrosis, (iv) 2 = moderately resistant = small to medium uredinia surrounded by chlorosis or necrosis, green island may be surrounded by chlorotic or necrotic border, (v) 3 = moderately susceptible = medium-sized uredinia that may be associated with chlorosis, (vi) 3 +/, 4 = susceptible = large uredinia without chlorosis). Infection types 0–3 were categorized as resistant, 4–5 as moderately resistant, 6–7 as moderately susceptible and 8–9 as susceptible. The scoring of the seedlings was carried out using the protocol as specified by Prasad et al., 2021.

### SNP genotyping and diversity analysis

To assess the underlying molecular diversity among the evaluated wheat genotypes, genomic DNA was extracted from young leaf tissues using CTAB method (Doyle and Doyle, 1990) with minor modifications. DNA quality and concentration were confirmed using a NanoDrop spectrophotometer and 1.0% agarose gel electrophoresis. High-quality DNA samples were subjected to SNP genotyping. Genotyping was performed using the DArTSNP KASP markers designed by CIMMYT, Mexico, as per below details.

### Statistical analysis

The phenotypic and genotypic data generated on the 20 mutants along with checks, was subjected to following statistical analysis:

Analysis of Variance (ANOVA): The ANOVA was carried out to assess the significance of the main effects of environment and genotype and their interaction effect on different traits. For genotype × environment interaction analysis, each crop season at the experimental site was considered as a separate environment: E1 = 2021–22, E2 = 2022–23, and E3 = 2023–24. The probability levels were kept to 1% and 5% to test the statistical levels of significance. Furthermore, minimum differences among the mean values were estimated *via* the least significant difference (LSD) test.

The PCA and cluster analyses were performed using NCSS 2020 software. Principal Component Analysis values were used to construct a dendrogram based on the Euclidean distance matrix. The mutant lines were grouped according to the Ward’s minimum variance method.

Additionally, GGE bi-plot analysis was performed for estimation of the degrees of association among traits and mutant lines and for the identification of suitable mutant lines with desirable trait combinations using the GGE bi-plot tool.

*Genotype plus genotype by environment interaction (GGE) biplot analysis:* This analysis was performed on the grain yield basis using the statistical software tool GenStat 64-bit version 18.1 according to the method described below:


Yij​−Yˉ.j​=λ1​ξi1​ηj1​+λ2​ξi2​ηj2​+ϵij​

where *Yij* is the mean for the *i^th^* genotype in the *j^th^* location/environment, *μ* is the overall mean, *λ1* and *λ2* are the singular values of the first and second principal components (PC1 and PC2) respectively, *ƭ1iᶯ* and *ƭ2iᶯ* are the eigenvectors for the *i^th^* genotype for PC1 and PC2respectively, (*1j* and *2j*) are the eigenvectors for the *j^th^* genotype for PC 1 and PC 2 respectively, and eij is the residual error term.

*AMMI analysis:* AMMI analysis is a statistical method used to assess genotype and environment effects in multi-environment trials. AMMI model helps in understanding and visualizing the complex genotype by environment interaction, allowing for the identification of stable genotypes across different environments and the assessment of crop yield stability.


$$Y_{ij}=\mu +G_{i}+E_{j}+\sum_{k=1}^{n}\lambda_{k}\alpha_{ik}\gamma_{jk}+\epsilon_{ij}$$

Where *Y_ij_* is the response variable obtained from the *i* th genotype in the *j* th environment; μ is the grand mean; *G_i_* and *E_j_* are the genotype and environment deviations from the grand mean, respectively; *λ_k_* is the eigenvalue of the PCA analysis axis k; *α_ik_* and *γ_jk_* are the genotype and environment principal component scores for axis k; n is the number of principal components retained in the model, and *ϵ_ij_* is the error term.

*Cultivar Superiority Index:* The cultivar superiority (P_i_) for grain yield was calculated to identify the most stable and superior mutant wheat genotypes across test environments (Lin and Binns, 1988).


$$Pi=\sum_{j=1}^{n}[\frac{{\left(Xij\;-\;Mj\right)}^{2}}{2n}]$$

Where P*i* = superiority index of the *i_th_* cultivar;
*X_ij_* = yield of the i-th cultivar in the *j_th_* environment;
*M_j_* = maximum response obtained among all the cultivars in the j-th environment;
*n* = number of environments

*Diversity analysis using DArT-SNP data:* Data were obtained from the LGC Genomics grid report and curated for downstream genetic diversity analyses. Genotype calls were cleaned and numerically coded as 0, 1 and 2 to represent the two homozygous and heterozygous genotype states, respectively. SNP markers with a call rate ≥90% were retained for downstream analyses, corresponding to a maximum of 10% missing genotype calls per marker. No additional MAF threshold was imposed, as the analysis was based on a pre-selected panel of trait-associated KASP markers. Quality-filtered SNP data were converted into VCF and GDS formats using the vcfR and SNPRelate packages in R for downstream population structure and diversity analyses. Data processing and diversity analyses were performed in R using the tidyverse, stringr, glue, pheatmap, factoextra, ggrepel, RColorBrewer and reshape2 packages, together with base R functions.

Principal component analysis (PCA) was performed using the prcomp function in Rafter removal of invariant markers. Missing genotype data were handled during preparation of the PCA dataset, and genotype variables were scaled prior to PCA. The resulting principal component scores were visualized using the factoextra package.

Pairwise IBS distance was estimated as the proportion of loci showing different genotype states between each pair of genotypes, considering the available genotype calls for the comparison. A value of 0 indicated identical genotype states, whereas increasing values represented greater genotype mismatch. The resulting pairwise IBS distance matrix was visualized as a heatmap using the pheatmap package. Hierarchical clustering was performed on the same distance matrix using the hclust function with complete-linkage clustering. Multidimensional scaling (MDS) was also performed using the IBS distance matrix to visualize relationships among genotypes. A genotype heatmap was generated using the base R heatmap function to visualize SNP-level genotype states across the evaluated genotypes.

## Results

The phenotypic variation observed among the irradiation-derived lines demonstrates the effectiveness of the mutagenesis-selection approach in generating useful breeding variation from the DBW88 genetic background. The selected mutants differed in phenology, plant architecture, yield components and yellow rust response, indicating that irradiation followed by phenotypic selection produced lines with contrasting agronomic and disease-resistance phenotypes. Importantly, several mutants combined improved rust resistance with desirable agronomic performance, demonstrating the practical value of induced variation for diversifying an otherwise elite and relatively uniform breeding background.

### Analysis of variance

Mean squares of the Univariate ANOVA were computed using the data recorded from the mutants and checks for different traits including yellow rust disease of wheat (**Table 2**). Few of the traits: DH, Tillers, SL showed significant differences (P<0.05 or P<0.01) among the genotypes across three crop seasons. The genotypes were evaluated for three years and observations were recorded for different traits namely days to heading, plant height, no. of tillers/m, spike length, no. of spikelets per spike, grain weight, grain no. per spike, grain weight per spike, thousand grain weight. The mean and range of each trait is presented in **Table 3**.

**Table 2:** Pooled ANOVA for different traits recorded from three-year data.

Source	df	DH	PH	T	SL	S	TSW
ENV	2	261.6736*	607.4811*	22537.22*	22537.22*	137.7901**	12856.23**
REP(ENV)	3	12.22917*	21.67324	2171.951**	2171.951**	1.885802	2.940299
GEN	23	13.891	21.08248	1164.753*	1164.753*	5.371846**	53.1344*
Residuals	69	4.40308	13.21087	361.7412	361.7412	1.570183	41.65594
CV (%)		2.117023	3.290968	15.44818	15.44818	5.826974	19.24101
Source	df	PSW	TnG	GW	GnPS	GwPS	TGW
ENV	2	40.50896**	1627099**	1510.932**	7763.941**	12.69446**	242759.1**
REP(ENV)	3	0.032427	3908.097	28.30681*	50.84747	0.299651	58.40243
GEN	23	0.676999	7499.975	14.87006	95.14197	0.223483	133.9139
Residuals	69	0.15667	3543.778	9.084124	55.11284	0.144762	43.4291
CV (%)		15.77984	14.62495	19.54746	13.99118	18.50246	8.500538

* Significant at 0.05% level of significance.

Means with the same letters are not statistically significant. Where, D, days to heading; PH, plant height (cm); T, No. of tillers/m; SL, spike length (cm); S, No. of spikelets; TSW, Total spikelet weight; PSW, Per spike weight (g); TnG, Total no. of grains; GW, Grain weight (g); GnPS, Grain no. per spike; GwPS, Grain weight per spike (g); TGW, Thousand grain weight (g).

**Table 3:** Mean values recorded on the mutants and checks for different across three years.

Genotypes	DH	PH	T	SL	S	TSW	PSW	TnG	GW	GnPS	GwPS	TGW
DTWM-01	99.83 ^abcd^	108.08 ^bc^	154.17 ^a^	13.82 ^de^	22.78 ^a^	33.06 ^abcd^	2.29 ^ab^	401.67 ^abcd^	14.42 ^a^	52.05 ^abcd^	1.91 ^ab^	69.15 ^e^
DTWM-02	98.67 ^bcde^	109.49 ^abc^	125.00 ^ab^	14.96 ^abcd^	20.44 ^cdef^	36.40 ^abcd^	2.66 ^ab^	401.67 ^abcd^	15.43 ^a^	53.52 ^abcd^	2.11 ^ab^	78.33 ^abcde^
DTWM-03	99.33 ^abcd^	111.47 ^ab^	106.33 ^b^	13.85 ^de^	20.67 ^bcdef^	40.81 ^a^	3.06 ^a^	424.50 ^abcd^	17.25 ^a^	56.86 ^abcd^	2.36 ^ab^	77.53 ^abcde^
DTWM-04	99.83 ^abcd^	111.81 ^ab^	111.67 ^b^	14.78 ^abcde^	21.89 ^abcde^	33.63 ^abcd^	2.46 ^ab^	429.83 ^abcd^	15.93 ^a^	56.43 ^abcd^	2.10 ^ab^	78.14 ^abcde^
DTWM-05	99.33 ^abcd^	109.61 ^abc^	129.33 ^ab^	13.97 ^de^	22.56 ^ab^	30.12 ^bcd^	2.16 ^b^	377.33 ^cd^	14.99 ^a^	48.94 ^d^	1.97 ^ab^	80.13 ^abcd^
DTWM-06	98.50 ^bcde^	112.63 ^ab^	117.83 ^b^	14.33 ^bcde^	22.11 ^abcd^	33.39 ^abcd^	2.28 ^ab^	397.67 ^abcd^	14.09 ^a^	52.46 ^abcd^	1.91 ^ab^	73.83 ^bcde^
DTWM-07	98.67 ^bcde^	110.49 ^abc^	120.00 ^ab^	14.07 ^cde^	21.56 ^abcdef^	38.92 ^ab^	2.90 ^ab^	379.50 ^cd^	16.35 ^a^	50.77 ^bcd^	2.23 ^ab^	80.15 ^abcd^
DTWM-08	99.83 ^abcd^	111.42 ^ab^	118.33 ^b^	14.90 ^abcde^	21.78 ^abcde^	32.30 ^abcd^	2.37 ^ab^	399.33 ^abcd^	13.99 ^a^	52.13 ^abcd^	1.89 ^ab^	74.18 ^bcde^
DTWM-09	98.17 ^cde^	108.61 ^abc^	128.08 ^ab^	13.86 ^de^	21.33 ^abcdef^	30.84 ^abcd^	2.57 ^ab^	378.17 ^cd^	15. 9 ^a^	48.95 ^d^	1.97 ^ab^	78.88 ^abcde^
DTWM-10	98.50 ^bcde^	109.96 ^abc^	112.50 ^b^	13.78 ^e^	22.11 ^abcd^	33.92 ^abcd^	2.50 ^ab^	395.50 ^bcd^	15.27 ^a^	51.46 ^bcd^	2.03 ^ab^	75.32 ^bcde^
DTWM-11	100.00 ^abcd^	112.53 ^ab^	114.25 ^b^	13.75 ^e^	22.33 ^abcd^	34.48 ^abcd^	2.70 ^ab^	359.00 ^d^	14.33 ^a^	48.38 ^d^	1.96 ^ab^	78.73 ^abcde^
DTWM-12	97.83 ^de^	112.04 ^ab^	125.08 ^ab^	14.19 ^cde^	21.78 ^abcde^	34.49 ^abcd^	2.42 ^ab^	385.83 ^cd^	13.75 ^a^	50.69 ^cd^	1.87 ^ab^	70.97 ^de^
DTWM-13	100.50 ^abcd^	110.23 ^abc^	110.08 ^b^	14.91 ^abcde^	21.67 ^abcde^	32.18 ^abcd^	2.41 ^ab^	423.67 ^abcd^	15.95 ^a^	54.69 ^abcd^	2.09 ^ab^	75.12 ^bcde^
DTWM-14	101.17^ab^	110.15 ^abc^	120.58 ^ab^	14.21 ^cde^	22.11 ^abcd^	31.62^abcd^	2.12 ^b^	362.00 ^d^	13.18 ^a^	47.84 ^d^	1.79 ^b^	72.77 ^bcde^
DTWM-15	98.83 ^abcde^	112.64 ^ab^	134.33 ^ab^	15.51^a^	21.11 ^abcdef^	38.02 ^abc^	2.86 ^ab^	403.67 ^abcd^	16.18 ^a^	53.43 ^abcd^	2.22 ^ab^	81.27 ^abcd^
DTWM-16	96.00 ^e^	108.11 ^bc^	119.58 ^ab^	15.39 ^ab^	19.56 ^f^	31.83 ^abcd^	2.88 ^ab^	401.50 ^abcd^	17.31 ^a^	51.32 ^bcd^	2.25 ^ab^	86.44 ^a^
DTWM-17	98.17 ^cde^	110.67 ^abc^	129.67 ^ab^	15.21 ^abc^	20.78 ^abcdef^	30.08 ^bcd^	2.50 ^ab^	419.50 ^abcd^	15.18 ^a^	53.47 ^abcd^	1.98 ^ab^	80.29 ^abcd^
DTWM-18	98.67 ^bcde^	109.77 ^abc^	117.25 ^b^	14.89 ^abcde^	20.33 ^def^	28.69 ^cd^	2.26 ^ab^	414.67 ^abcd^	15.88 ^a^	51.78 ^bcd^	2.06 ^ab^	81.62 ^abc^
DTWM-19	99.00 ^abcd^	112.61 ^ab^	130.92 ^ab^	14.98 ^abcd^	21.78 ^abcde^	39.25 ^ab^	2.88 ^ab^	456.33 ^abc^	17.94 ^a^	59.73 ^abc^	2.41 ^a^	77.86 ^abcde^
DTWM-20	101.67^a^	113.21 ^a^	123.17 ^ab^	14.13 ^cde^	22.44 ^abc^	33.77 ^abcd^	2.39 ^ab^	436.17 ^abcd^	15.47 ^a^	55.97 ^abcd^	2.05 ^ab^	71.41 ^cde^
WH1105 (C)	98.67 ^bcde^	109.69 ^abc^	128.58 ^ab^	15.22 ^abc^	20.78 ^abcdef^	34.29 ^abcd^	2.66 ^ab^	480.17 ^ab^	17.47 ^a^	61.71 ^a^	2.30 ^ab^	81.91 ^ab^
DBW88 (C)	97.83 ^de^	106.1556 ^c^	118.92 ^b^	13.87 ^de^	21.89 ^abcde^	31.82 ^abcd^	2.37 ^ab^	481.17 ^a^	16.98 ^a^	60.47 ^ab^	2.17 ^ab^	77.44 ^abcde^
HD3086 (C)	98.83 ^abcde^	110.15 ^abc^	138.67 ^ab^	14.41 ^abcde^	20.00 ^ef^	27.76 ^d^	2.30 ^ab^	381.67 ^cd^	14.42 ^a^	49.74 ^d^	1.89 ^ab^	81.44 ^abc^
HD2967 (C)	101.00^abc^	109.16 ^abc^	120.50 ^ab^	14.51 ^abcde^	22.33 ^abcd^	33.38 ^abcd^	2.20 ^ab^	378.50 ^cd^	13.23 ^a^	50.68 ^cd^	1.81 ^ab^	77.72 ^abcde^
Mean	99.11806	110.444	123.1181	14.4787	21.50463	33.54368	2.51	407.04	15.419	53.061	2.056	77.53
CV	1.43	2.179508	13.61858	3.910068	4.601123	14.49158	17.02	10.06	15.298	8.891	14.106	6.53
t-value	2.068658	2.068658	2.068658	2.068658	2.068658	2.068658	2.07	2.07	2.069	2.069	2.069	2.07
LSD 5%	2.931953	4.979539	34.68504	1.171123	2.046842	10.05576	0.88	84.74	4.880	9.759	0.600	10.47

Means with the same letters are not statistically significant. Where, DH= days to heading, PH= plant height (cm), T= No. of tillers/m, SL= spike length (cm) S= No. of spikelets, TSW= Total spikelet weight, PSW= Per spike weight (g), TnG= Total no. of grains, GW= Grain weight (g), GnPS= Grain no. per spike, GwPS= Grain weight per spike (g), TGW=Thousand grain weight (g).

### Superiority index of grain yield

The superiority index (P_i_), proposed by Lin and Binns (1988) was used to identify the most stable and superior wheat genotypes for grain yield performance across test environments. Genotypes with low Pi values are useful candidates when the breeding objective is consistent yield performance across contrasting environments, whereas genotypes showing superior performance specifically in favorable or unfavorable environments may be useful for targeted adaptation. Across the environments, the P_i_ values ranged from 0.14 to 2.28, indicating a considerable variation among the genotypes (**Table 4**). Mutant DTWM-16 (P_i_ = 0.14) recorded the lowest value of superiority index, followed by WH1105 (P_i_ = 0.19) and DTWM-15 (Pi = 0.35), signifying their superior and stable yield performance across environments. In contrast, DTWM-12 (P_i_ = 2.28) and DTWM-20 (P_i_ = 2.07) exhibited relatively higher P_i_ values, indicating greater deviation from the maximum yield and thus lower stability.

**Table 4:** The superiority index of grain yield for wheat genotypes.

GEN	Y	Pi_a	R_a	Pi_f	R_f	Pi_u	R_u
DTWM-01	2.912333	1.09095	17	1.2458	23	0.78125	11
DTWM-02	2.5425	1.707446	18	1.456042	24	2.210253	17
DTWM-03	3.035	0.928187	14	0.744256	19	1.29605	14.5
DTWM-04	3.194667	0.621845	10	0.566742	13	0.73205	9
DTWM-05	3.411333	0.45573	6	0.302289	7	0.762613	10
DTWM-06	2.595667	1.749867	19	0.7204	17	3.8088	19
DTWM-07	2.959333	1.075827	16	0.965715	22	1.29605	14.5
DTWM-08	3.617	0.363303	5	0.544954	12	0	1
DTWM-09	3.288167	1.001726	15	0.119025	3	2.767128	18
DTWM-10	3.078333	0.785029	13	0.738637	18	0.877813	12
DTWM-11	3.072	0.772727	12	0.68299	15	0.9522	13
DTWM-12	2.610667	2.288415	24	0.295295	6	6.274653	24
DTWM-13	2.758	1.820014	20	0.284708	5	4.890628	21
DTWM-14	2.674	1.984754	21	0.385031	9	5.1842	22
DTWM-15	3.480833	0.353798	3	0.448759	10	0.163878	5
DTWM-16	3.8005	0.141981	1	0.194407	4	0.037128	2
DTWM-17	3.496167	0.358452	4	0.376677	8	0.322003	6
DTWM-18	3.391167	0.562353	8	0.796765	21	0.093528	3
DTWM-19	3.224167	0.59806	9	0.715401	16	0.363378	7
DTWM-20	2.473667	2.072194	22	0.775035	20	4.666513	20
WH1105 (C)	3.860667	0.198971	2	0.017556	1	0.5618	8
DBW88 (C)	3.4945	0.750964	11	0.026569	2	2.199753	16
HD3086 (C)	3.3845	0.456485	7	0.605625	14	0.158203	4
HD2967 (C)	2.527833	2.241003	23	0.48424	11	5.754528	23

GEN, genotype; Y, grain yield; Pi_a, superiority measure for all environments; R_a, rank for Pi_a; Pi_f, superiority measure for favorable environments; R_f, rank for Pi_f; Pi_u, superiority measure for unfavorable environments; R_u, rank for Pi_u.

### Favorable and unfavorable environment?

When environments were partitioned, the Pi_(f) values for favorable environments identified WH1105, DBW88 and DTWM-09 as the best performers, while the Pi_(u) values for unfavorable environments indicated DTWM-08 and DTWM-16 as the most adaptable under stress conditions. This suggests that these genotypes possess specific adaptation potential.

The results of the superiority index are consistent with the AMMI and GGE biplot analyses, confirming that genotypes with low Pi values maintained stable performance and can be considered potential candidates for multi-environment stability and high yield in wheat breeding programs.

### Pearson correlation coefficient

The correlation analysis revealed that grain yield was positively and significantly associated with grain number per spike, grain weight, total number of grains, and number of spikes, while it showed a significant negative association with days to heading and spike length. These results indicate that yield improvement can be achieved by selecting genotypes with higher spike productivity and grain weight coupled with early heading behavior.

The Pearson’s correlation coefficients were analyzed to understand the relations between various traits (**Figure 1**). Significant positive correlation was observed for yield with GNPS (0.55***), GW (0.45***), SL (0.27***) and negative correlation with NS (−0.55***), DTH (−0.54***).

**Figure 1:**
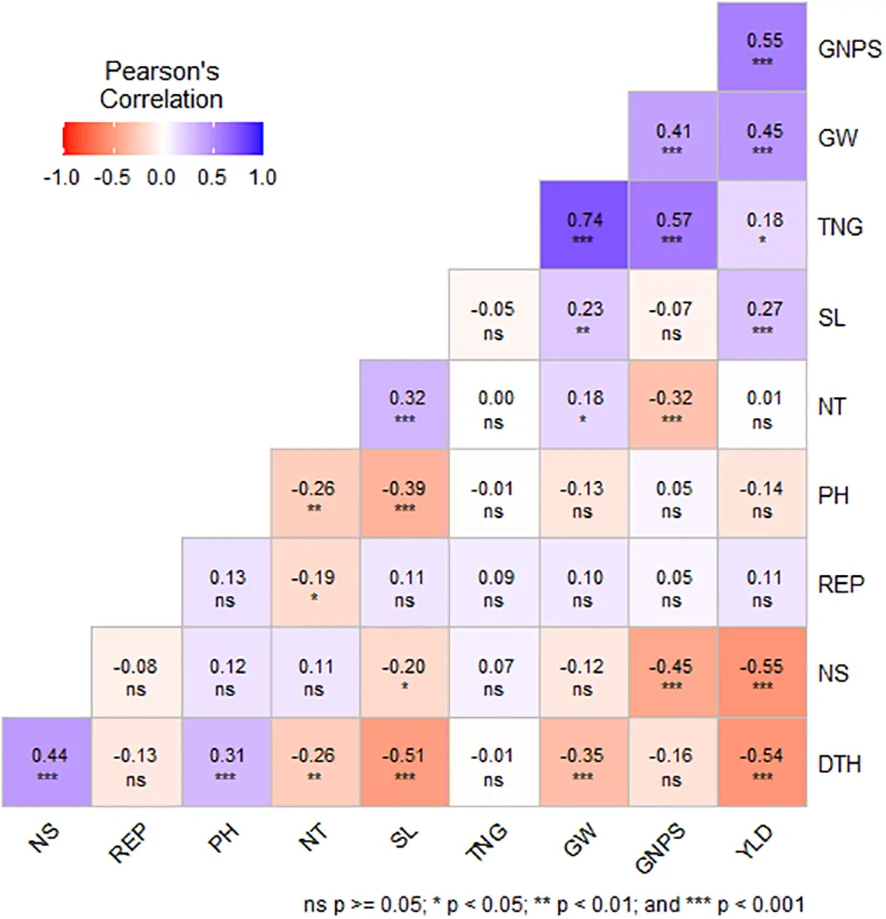
Pearson correlation matrix showing the associations among grain yield and yield-related agronomic traits of wheat mutant genotypes evaluated across three crop seasons.

Yellow rust infection was severe across season, allowing for clear and reliable scoring of field reactions. A diverse range of field reactions to yellow rust, such as immune (I), resistance (R), moderately resistance (MR), moderately resistant to moderately susceptible provides some degree of protection (MRTR), susceptible (S) was observed among mutants and standards. Eight genotypes were found immune (DTWM-03, DTWM-08, DTWM-10, DTWM-11, DTWM-16, DTWM-18, DTWM-19, HD3086) while 42% were susceptible, 8% were displayed R, MRTR, MR and DBW88 parent was found highly susceptible.

The correlation analysis among Average Coefficient of Infection (ACI), Grain Weight (GW), Thousand Grain Weight (TGW), and Total Number of Grains (TNG) was performed using Pearson’s correlation (**Table 5**) in R Studio, and the results are presented in the correlation plot (**Figure 2**). The corrplot clearly shows the interrelationships between the studied traits, where color intensity and size of circles represent the strength and direction of correlation values.

**Table 5:** Coefficient of Infection and yellow rust response in mutants, standard checks and parent.

Gen	Adult plant resistance	Average coefficient of infection	Grain weight (g)	TGW	TNG
DTWM-01	40S	40	12.995	45.1224	289
DTWM-02	33.3S	33.3	3.855	18.91765	203
DTWM-03	0R	0	16.1	43.31259	371.5
DTWM-04	20MRTR	1.6	11.88	37.00249	322.5
DTWM-05	10 MR	4	13.48	43.8906	305
DTWM-06	26.6S	26.6	7.875	25.23996	312
DTWM-07	10MRTR	0.8	15.005	41.86765	358
DTWM-08	OR	0	13.485	41.56169	323.5
DTWM-09	10MR	4	12.92	46.44893	277.5
DTWM-10	0R	0	11.615	42.42174	273
DTWM-11	0R	0	14.875	45.34303	328
DTWM-12	40S	40	6.37	24.63456	217
DTWM-13	66.6S	66.6	7.005	26.7932	258
DTWM-14	33.3S	33.3	5.935	23.69939	255.5
DTWM-15	5R	1	14.675	44.49989	329
DTWM-16	0R	0	15.64	53.0971	294.5
DTWM-17	5R	1	16.47	44.2808	372
DTWM-18	0R	0	15.905	53.0233	299.5
DTWM-19	0R	0	16.265	46.15213	352
DTWM-20	30S	30	6.935	23.70998	294.5
WH1105 (C)	13.3S	13.3	15.27	35.73946	428.5
DBW88 (C)	33.3S	33.3	9.64	31.0706	309.5
HD3086 (C)	0R	0	16.91	45.64324	371
HD2967 (C)	53.3S	53.3	4.74	19.22444	246.5

**Figure 2:**
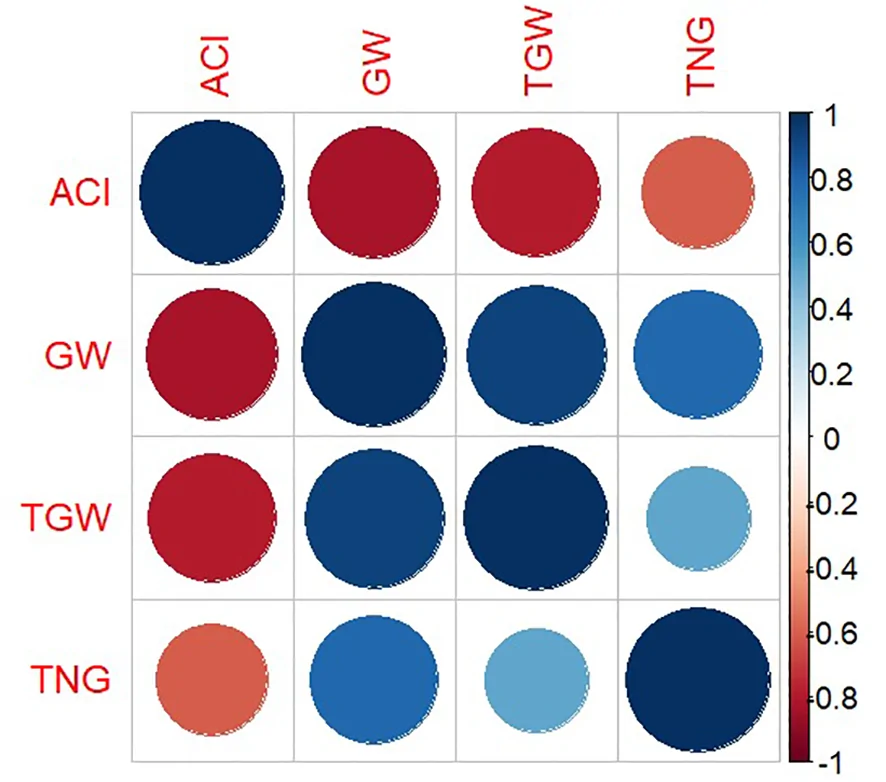
Correlation between ACI for stripe rust and grain traits.

A negative correlation was observed between ACI and all yield-related traits (GW, TGW, and TNG), indicating that an increase in stripe rust infection severity leads to a decline in yield performance. This is visually evident by the red-shaded circles in the matrix. Among these, ACI and TGW exhibited the strongest negative relationship, suggesting that rust infection significantly reduces grain size and weight. Conversely, strong positive correlations were observed among the yield components (GW, TGW, and TNG), as shown by dark blue circles. This indicates that plants producing heavier grains tend to also have higher total grain numbers and overall yield. The highest positive correlation was observed between TGW and TNG, confirming their mutual contribution to yield enhancement. Overall, these findings demonstrate that rust severity adversely affects yield parameters, while yield traits themselves are positively interlinked. Hence, breeding for rust resistance (low ACI) could play a critical role in improving both grain weight and total yield in wheat mutants. To confirm the yellow rust resistance of the mutants and the checks, the set was screening at seven hotspot locations in the northern India, and the yellow rust reaction data is presented in **Table 6**.

**Table 6:** Screening of the mutants at hotspot locations for stripe rust, during 2024-25.

SN	Genotype	Gurdaspur	Dhaulakuan	Malan	Karnal	Durgapura	Hisar	Jammu	ACI	HS
1	DTWM-01	20S	0	20MS	10MR	10MS	10S	0	8.3	20S
2	DTWM-02	40S	10S	40S	20S	40S	20S	tMS	24.4	40S
3	DTWM-03	0	0	40S	10S	0	5S	0	2.1	40S
4	DTWM-04	20S	0	20MS	10MR	10S	5S	20MS	10.4	20S
5	DTWM-05	40S	0	10MS	20S	10S	5S	10MS	13.0	40S
6	DTWM-06	40S	0	40S	40S	20S	20S	0	22.9	40S
7	DTWM-07	20S	0	20MS	10S	10S	5S	0	8.7	20S
8	DTWM-08	0	0	10MS	10S	0	0	0	2.6	10S
9	DTWM-09	20S	0	40S	10MS	5S	0	tR	10.5	40S
10	DTWM-10	20S	0	40S	10S	20S	5S	tMS	13.7	40S
11	DTWM-11	20S	10S	40S	10S	10S	5S	20MS	15.9	40S
12	DTWM-12	40S	0	20MS	40S	20S	5S	10S	18.7	40S
13	DTWM-13	40S	20S	20MS	20S	40S	5S	10S	21.6	40S
14	DTWM-14	40S	20S	40S	40S	20S	5S	5MS	24.1	40S
15	DTWM-15	0	0	5MS	5S	40S	20S	0	9.9	40S
16	DTWM-16	0	0	5MS	5S	5S	20S	0	4.9	20S
17	DTWM-17	5S	0	5MS	5S	0	15S	0	4.1	5S
18	DTWM-18	5S	0	0	0	5S	15S	0	3.6	15S
19	DTWM-19	tS	0	5MS	0	10S	15S	20S	7.1	20S
20	DTWM-20	40S	10S	20MS	40S	40S	15S	10S	24.4	40S
21	WH1105 (C)	20S	0	60S	10S	20S	0	5MS	16.3	60S
22	DBW88 (C)	80S	0	40S	20S	20S	15S	5MS	25.6	80S
23	HD3086 (C)	10S	0	10MS	5S	0	15S	0	5.4	15S
24	HD2967 (C)	80S	10S	20MS	20S	40S	15S	tMS	26.0	80S

#### Seedling resistance test

The seedling resistance test results revealed a wide spectrum of wheat-pathotype interactions, ranging from nearly immune reactions to completely susceptible, thereby reflecting the diverse genetic makeup of genotypes (**Table 7**).

**Table 7:** Seedling Resistance test (SRT), for yellow and leaf rust pathotypes.

Genotypes	Stripe rust				Leaf rust			
	110S119	238S119	110S84	T	77-5	77-9	104-2	12-5
DTWM-01	0	1	7	0	2	9	2	1
DTWM-02	0	6	5	0	3	1	1	8
DTWM-03	0	9	5	0	1	1	2	1
DTWM-04	9	1	0	0	–	9	2	1
DTWM-05	0	0	5	0	2	9	1	1
DTWM-06	0	1	0	0	1	1	1	1
DTWM-07	0	1	0	0	9	9	1	0
DTWM-08	0	0	0	0	1	1	2	1
DTWM-09	0	0	0	0	5	9	2	2
DTWM-10	1	-	0	0	9	9	1	2
DTWM-11	0	9	0	0	9	9	1	2
DTWM-12	9	7	9	9	1	9	9	1
DTWM-13	-	-	0	-	2	1	9	1
DTWM-14	0	1	0	0	9	9	4	1
DTWM-15	0	1	0	0	9	9	2	1
DTWM-16	9	0	9	0	9	9	2	1
DTWM-17	7	9	5	9	9	1	2	1
DTWM-18	9	9	9	-	9	1	2	1
DTWM-19	9	9	0	9	9	1	5	1
DTWM-20	9	9	0	-	1	9	2	1
WH1105 (C)	9	9	9	0	9	1	1	2
DBW88 (C)	0	0	5	-	9	9	2	1
HD3086 (C)	5	0	0	0	9	9	9	1
HD2967 (C)	0	0	0	-	9	1	1	9

Among the evaluated mutants, DTWM-08 and DTWM-16 were particularly noteworthy because their disease-resistance profiles were accompanied by favorable agronomic performance. DTWM-08 showed a highly resistant adult-plant response and low average coefficient of infection, together with strong performance under unfavorable environments. DTWM-08 maintained a low disease response across the multi-location hotspot evaluation, although some site-specific variation in reaction was observed. DTWM-16 combined complete adult-plant resistance with a low superiority index and consistently favorable yield performance across environments. These lines therefore represent promising materials for simultaneous improvement of yield performance and stripe rust resistance, although their wider adaptation and durability of resistance require further multi-location validation.

### GE analysis by AMMI model

The AMMI model was used to assess genotype, location (environment), and genotype by location interaction. This model is a powerful analytical tool for genotype x environment studies that allows for the identification of adaptation targets, adaptive traits, and test sites. The AMMI model considers both the main effects and interaction effect of the factors, making it useful for identifying genotypes with desirable traits across different environments (**Table 8**). The ANOVA for grain yield using the AMMI method showed that Bread wheat grain yield is significantly (p < 0.01) affected by the main effects of environment (E) and genotype (G), and Genotype × Environment (G × E) interaction (**Table 8**). The first principal component is significant, while the second one is not.

**Table 8:** The variance of AMMI analysis of grain yield for wheat genotypes.

Source	Degree of freedom	Sum of squares	Mean squares	F value	Pr(>F)
ENV	2	75.80883	37.90441	20.50416*	0.017798
REP(ENV)	3	5.545863	1.848621	4.931477*	0.003673
GEN	23	23.94075	1.040902	2.776765*	0.000577
GEN: ENV	46	50.51227	1.098093	2.92933*	2.65E-05
PC1	24	40.69875	1.69578	4.52*	0
PC2	22	9.81352	0.44607	1.19	0.2856
Residuals	69	25.86544	0.374861		
Total	189	232.1854	1.228494		

* Significant at 1% level of significance.

#### AMMI1 biplot analysis

AMMI1 analysis revealed that IPCA1 explained 80.6% of the total G×E interaction, indicating a strong interaction pattern. In AMMI1 biplot, X-coordinate represents the main effects, which are the genotypes and environment mean, while the Y-coordinate represents the interaction effect, which is the IPCA1.

According to the AMMI1 biplot (**Figure 3**), genotype WH1105 had highest mean yield followed by DTWM-16, DTWM-08, DBW88, DTWM-17 and DTWM-15. WH1105 and DBW88 showed high mean yield but were specifically adapted to environment E2. Genotypes DTWM-04 and DTWM-05 were the most stable genotypes with an IPCA1 score close to zero compared to the other genotypes. Although, the genotypes DTWM-03, DTWM-07 and DTWM-02 were closed to the origin and stable, but their mean yields were on the lower side. The genotypes like DTWM-12, DTWM-14, HD2967 and DTWM-13 had high values of IPCA1 and low yield and were considered as not stable. In conclusion genotype best for E2 are DBW88, DTWM-17, DTWM-16, DTWM-15, DTWM-08 while for E1 (low yield environment) DTWM-12, DTWM-14, DTWM-13, HD2967, DTWM-20 are best, and DTWM-03, DTWM-05, DTWM-07, DTWM-11 are stable and widely adapted. Environment E1 was highly discriminating due to its large negative IPCA1 score. Environment 2 was high yielding with high additive genotypic main effects.

**Figure 3:**
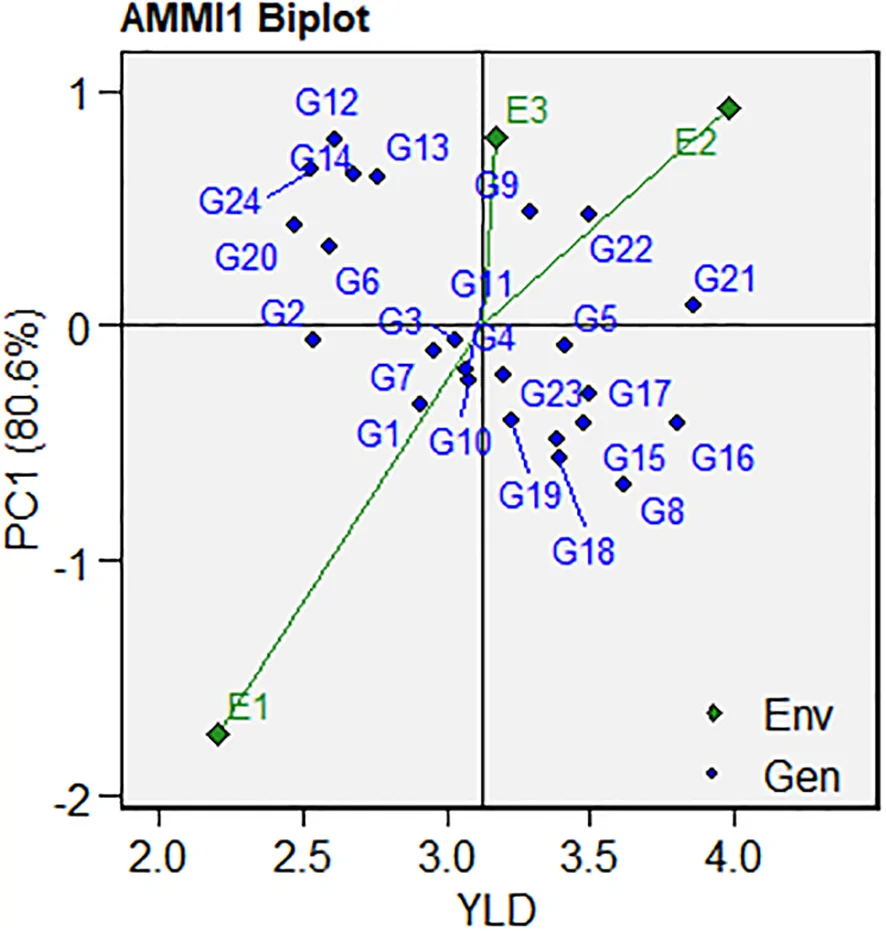
AMMI biplot model showing relationships among test environment and genotypes based on grain yield.

#### AMMI2 biplot analysis

AMMI2 biplot (**Figures 4a–c**) uses PC1 and PC2 scores simultaneously, which together explain the GxE interaction pattern. In AMMI-II biplot, first two principal components were significant and explains 100% of the variation. Environment scores in biplot are joined to the origin by vector. Genotypes which are near to origin are highly stable, while those far from origin has pronounced environmental effect, more adapted to specific environment, genotype having short vector distance are best adapted across all environment. According to the AMMI-II biplot, DTWM-09, DTWM-13, DTWM-12 and DTWM-20 had a positive interaction with E3; DTWM-02, DTWM-03, DTWM-07, DTWM-14, and HD2967 in E2; HD3086, DTWM-15, DTWM-19 E1. Same direction of the genotype and environment from origin represents positive interaction. The genotypes DTWM-18, DTWM-12, DTWM-01 and DTWM-14 located farthest from the biplot origin, contributed the largest G-E interaction for yield. Genotype HD2967, DTWM-14, DTWM-12, DBW88 and DTWM-13 were clustered with E2 and E3, indicating that these genotypes are stable in the respective environments. Genotypes DTWM-04, DTWM-11, DTWM-15, DTWM-16, DTWM-19, WH1105 were consistent across environments i.e., stable performers.

**Figure 4:**
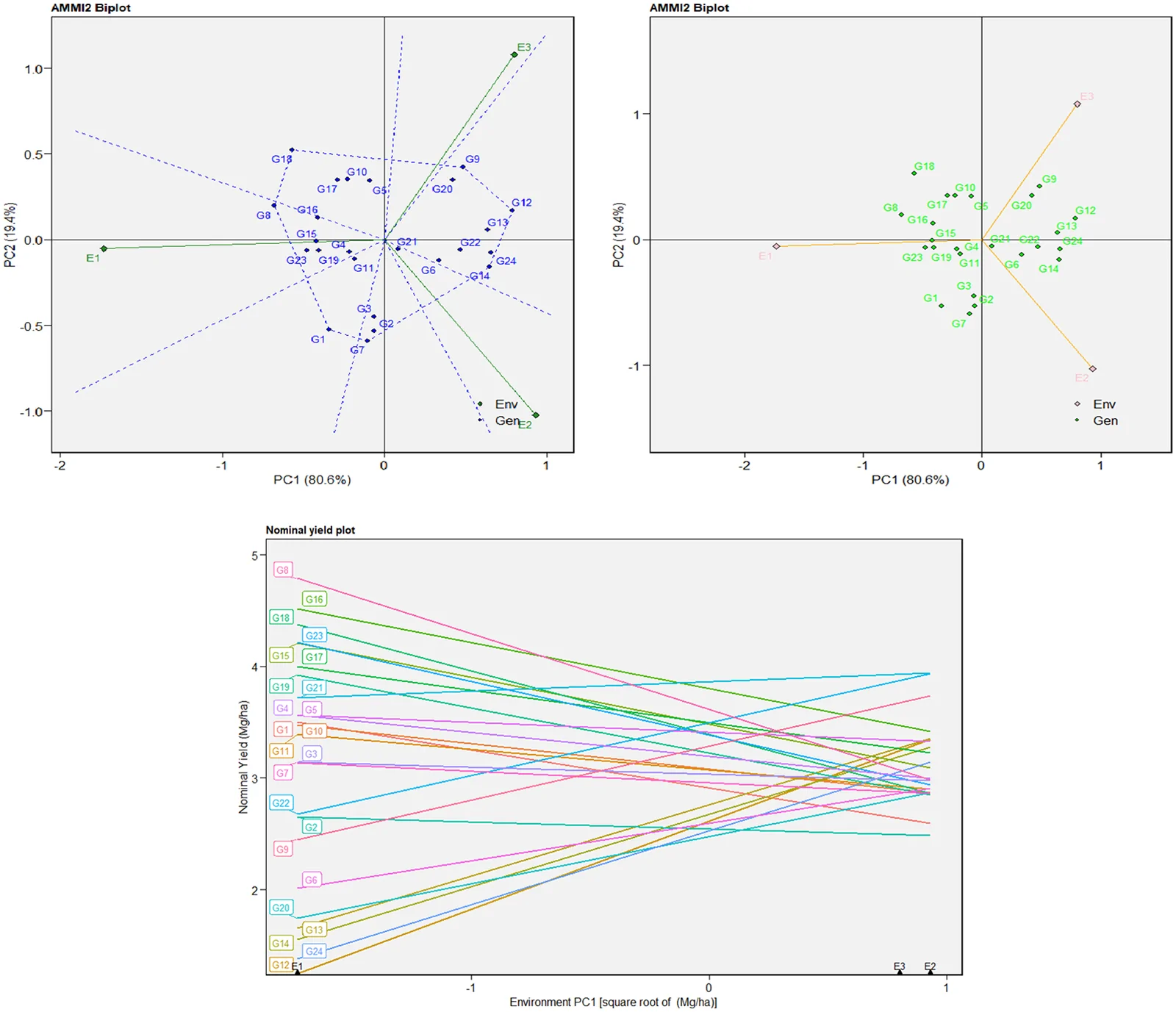
AMMI biplot model showing relationships among **(a)** IPC1 and IPC2 (polygon view) **(b)** IPC1 and IPC2 **(c)** Yield and Environment.

In **Figure 4c** high yielding and stable genotypes are DTWM-16, DTWM-18, DTWM-23, DTWM-15, DTWM-17, average yielding but stable genotypes are DTWM-04, DTWM-05, DTWM-11, DTWM-19, WH1105, while genotypes showing high yield in E3 are DTWM-09, DTWM-12, DTWM-13, DTWM-20, and in E2 are DTWM-02, DTWM-03, DTWM-07, DTWM-14, HD2967, and DTWM-06, DTWM-12, DTWM-13, DTWM-14, DTWM-20, HD2967 are poor yielders.

### GE analysis GGE biplot analysis

The most stable genotype for each environment was determined using the GGE biplot analysis. In the present study “Which-Won-Where” biplot divided by equality lines into eight sectors (**Figure 5**). The genotypes DTWM-12, DTWM-09, WH1105, DTWM-16, DTWM-08, DTWM-01, DTWM-02, and HD2967 were located on the vertices of the polygon. These genotypes are either the best or the poorest across different environments. The winning genotypes were DTWM-08 and DTWM-16 in environment 1 (E1), WH1105 and DTWM-09 in environment 3 (E3), and DTWM-12 and HD2967 in environment 2. The GGE biplot enables an evaluation of environments due to the discriminative ability and representativeness of the GGE view (Yan et al., 2007; Aktas, 2016).

**Figure 5:**
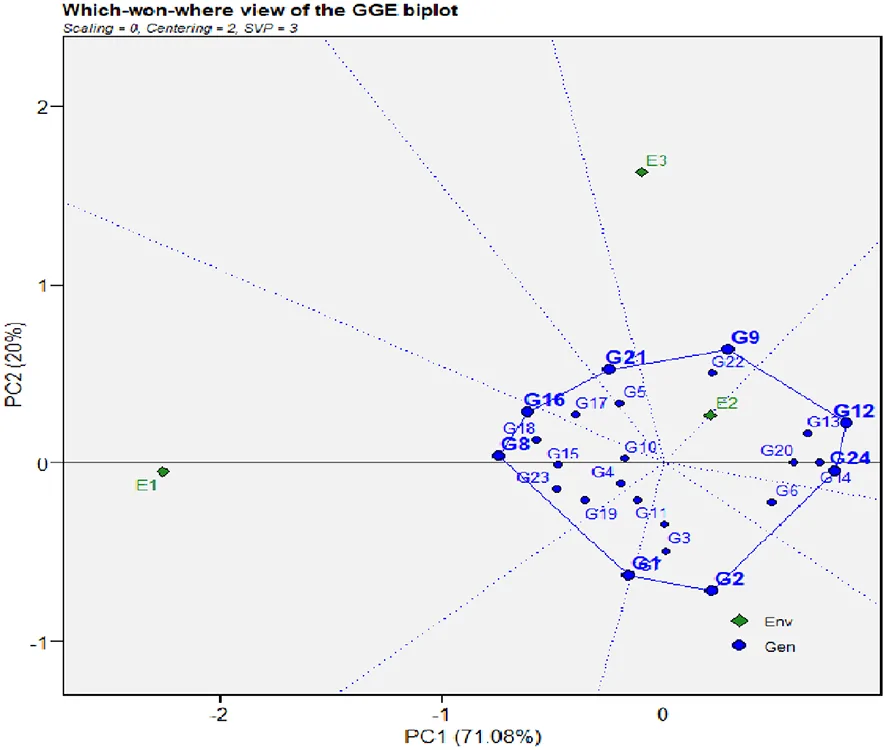
GGE biplot showing a) “which-won-where” pattern for genotypes and environments.

### Discriminativeness and representativeness

The discriminativeness vs representative GGE Biplot enables to recognize the favorable environments having highest discriminatory power to genotypes. Discriminativeness tells ability of environment to differentiate among genotypes and Representativeness tells how close the environment is to the average environment. Environment E1 is far from origin having high discriminativeness (long vector length) and less representativeness (less angle with AEC average environment coordinates). Environment E2 has high representativeness but less discriminativeness. Environment E3 has moderate discriminativeness and representativeness, hence a good test environment. So, genotypes DTWM-04, DTWM-11, DTWM-15, DTWM-16, DTWM-19, WH1105 are stable genotypes. Genotype showing specific adaptation to E1 are DTWM-08, DTWM-18, HD3086, for E2 are DTWM-04, DTWM-05, DTWM-15, DTWM-16, for E3 are DTWM-09, DTWM-12, DTWM-13, DTWM-20 (**Figure 6**).

**Figure 6:**
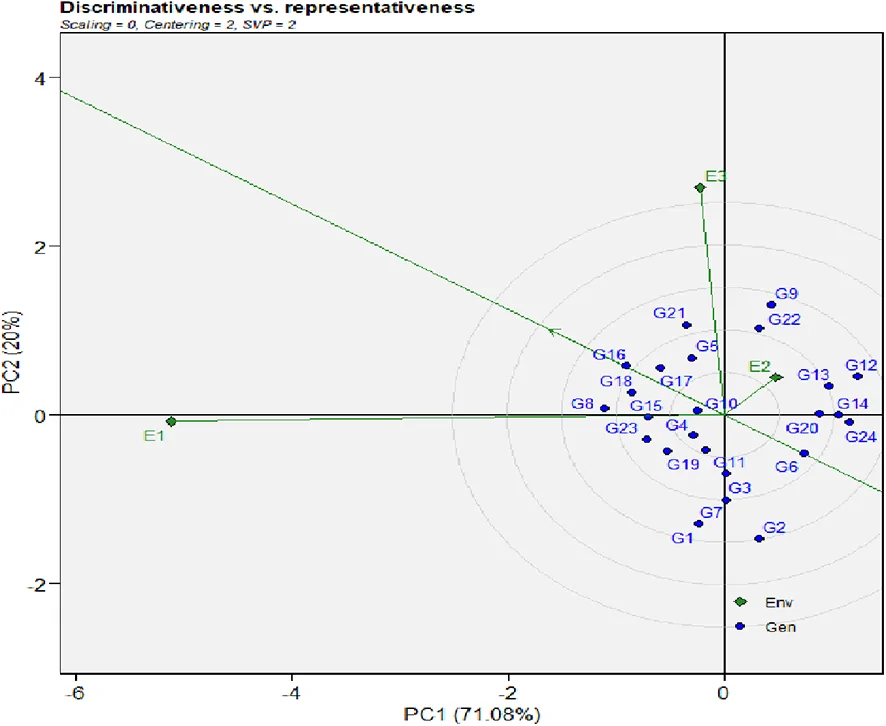
GGE biplot showing **(b)** discriminativeness & representativeness of environments for yield.

### Ranking of genotypes based on mean yield and stability performance

The genotype closer to the concentric circle produce more grain on average. The single arrowed line (**Figure 7**) in the present study indicates high yields in all locations/environments. The genotype with highest mean yield was DTWM-12, followed by DTWM-13>DTWM-09>DBW88>HD2967. The average yield for DTWM-10 was the lowest and the average yield for DTWM-11, DTWM-4, WH1105, DTWM-19 were closest to overall average. Additionally, DTWM-06, DTWM-02, DTWM-03, HD2967, DTWM-12, DTWM-13 were unstable across locations/environments as those are far away from average environment axis (AEC), while DTWM-11, DTWM-04, WH1105, DTWM-05, DTWM-17, DTWM-18, HD2967 were considered stable in that order.

**Figure 7:**
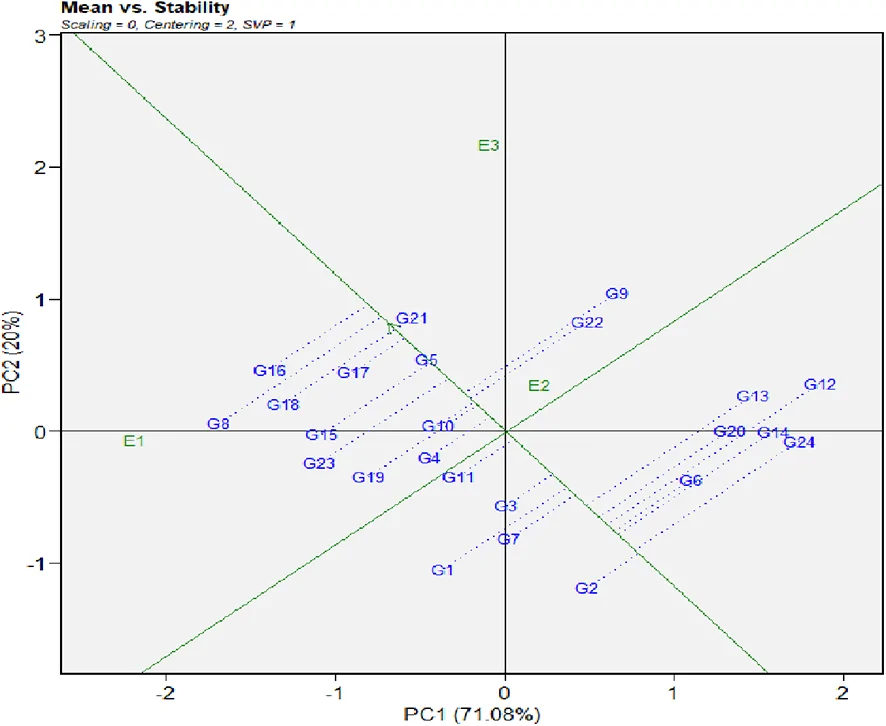
GGE biplot showing c) relationship between mean grain yield & wheat stability.

### Genetic diversity and population structure

#### Principal component analysis

Principal Component Analysis is a statistical method used to reduce the dimensionality of data while retaining most of the variation present in the original variables. PCA scores help summarize complex, multidimensional traits or genetic markers into key axes (PC1, PC2, PC3), allowing easier visualization and interpretation of similarities or differences among samples. Principal Component Analysis (PCA) based on 15 SNP markers clearly separated the wheat genotypes according to their genetic relationships (**Figure 8**) PC1, PC2, and PC3 represent the axes explaining the highest variance in the dataset. PC1 and PC2, PC1 and PC3 (fig) are used to plot the two-dimensional PCA biplot, which visually groups genetically similar individuals. The first two principal components (PC1 and PC2) captured the majority of the molecular variance, distinguishing a main cluster comprising most mutants and check varieties from a few divergent genotypes. Mutant lines DTWM-01 to DTWM-20 grouped closely together near the origin of the plot (PC1 ≈ 0.28; PC2 ≈ 0.22), indicating high genetic similarity. In contrast, mutants DTWM-10 and DTWM-14 were positioned far from the cluster along PC2 and PC1, respectively, representing genetically distinct individuals with novel allelic combinations. The parent (DBW88) and standard checks (WH1105, HD3086, HD2967) were located close to the main mutant group, confirming that induced mutagenesis produced subtle but detectable genomic variation without major departure from the parental background. Overall, the PCA results demons of the got rate that while most mutants retained parental genetic structure, a few lines exhibited substantial divergence that may contribute to phenotypic differentiation.

**Figure 8:**
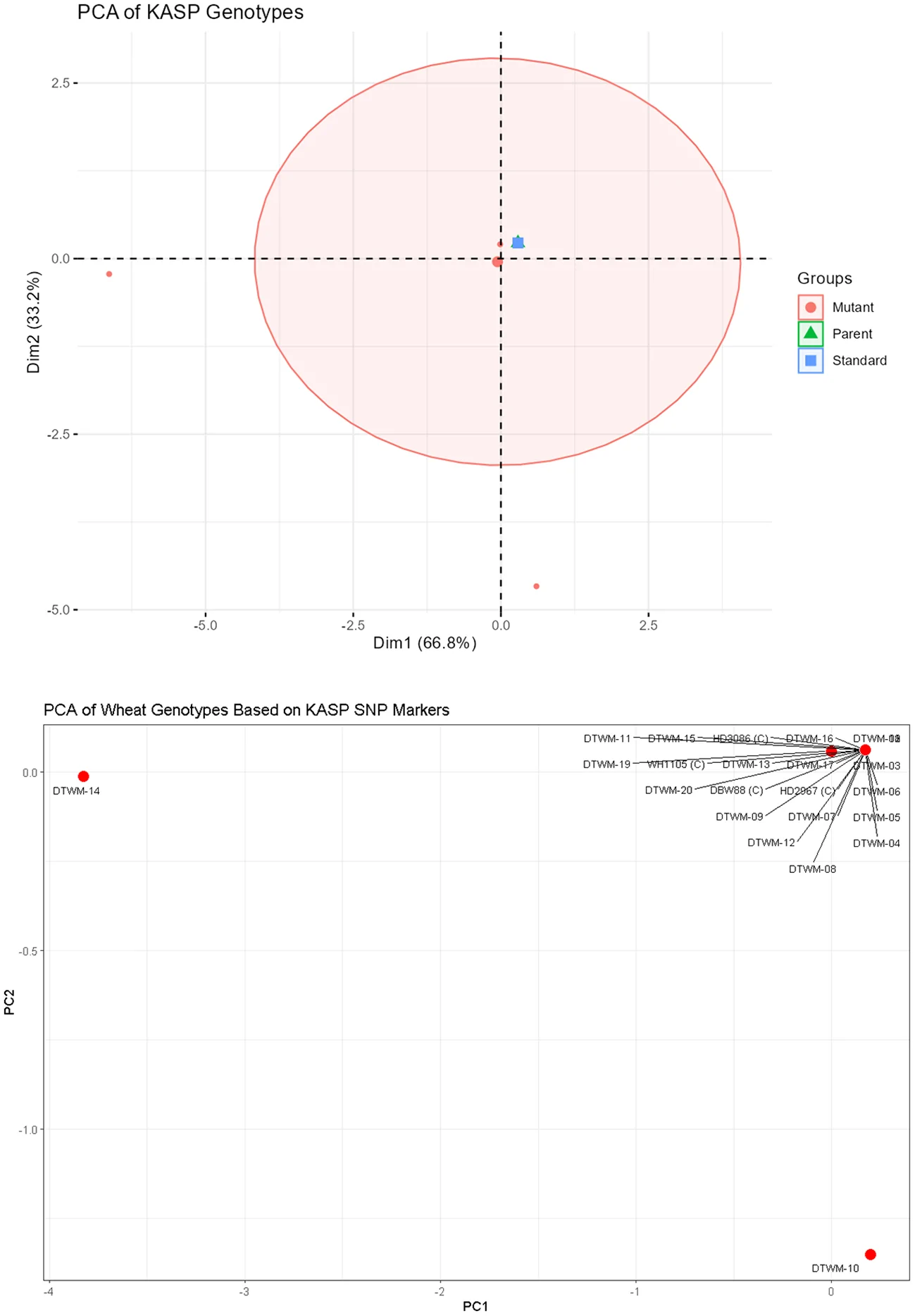
(1) Principal Component Analysis (PCA) plot showing the genetic relationships among wheat genotypes (mutants, parental, and standard checks) based on KASP SNP markers. (2) PC1-PC2 plot showing DTWM-14 and DTWM-10 to have unique allelic combinations.

The first two principal components (PC1 = 28%, PC2 = 22%) explain the majority of the molecular variance. Mutant lines (DTWM-01 to DTWM-20) cluster tightly near the origin, indicating high genetic similarity, whereas mutants DTWM-10 and DTWM-14 appear as outliers, reflecting distinct genetic divergence. The parental genotype (DBW88) and standard checks (WH1105, HD3086, HD2967) cluster near the main mutant group, suggesting limited but detectable differentiation from the parental genome.

### Identity by State heatmap

Pairwise IBS analysis (**Figure 9**) visualized genetic relatedness among the genotypes. Color intensity denotes similarity values ranging from 0 to 1. Most lines exhibited low IBS values (0.00-0.10), confirming a narrow genetic base among mutant lines. By contrast, DTWM-10 and DTWM-14 recorded higher IBS distances (0.20-0.25) from the remaining genotypes, reinforcing their genetic divergence and aligning with the dendrogram grouping.

**Figure 9:**
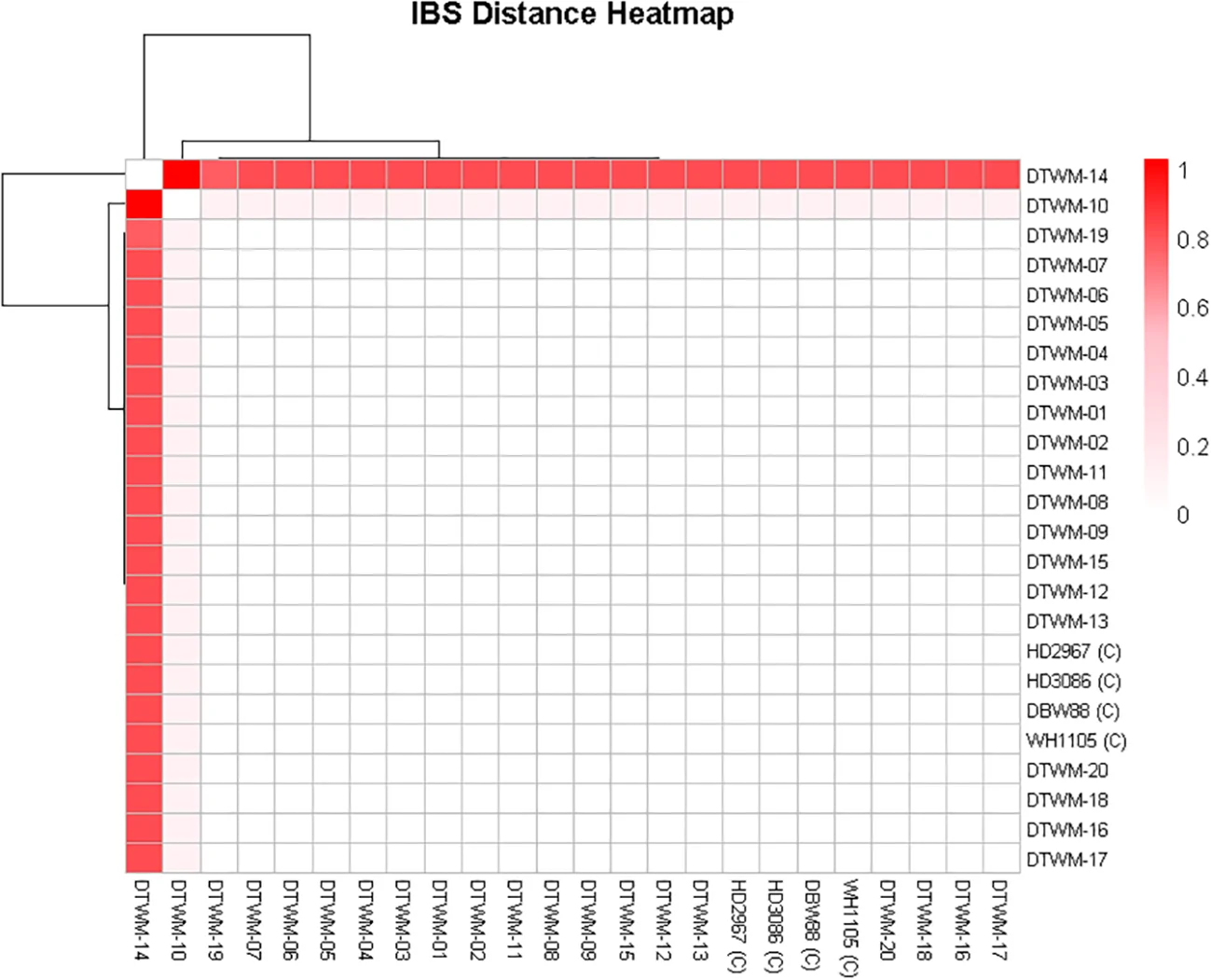
IBS heatmap showing genetic relatedness among the genotypes.

### Hierarchal clustering

The hierarchical clustering dendrogram (**Figure 10**) visually represents the genetic relationships among the genotypes analyzed in this study. Genotypes such as “DTWM-14 Mutant” and “DTWM-10 Mutant” form distinct clusters at higher dendrogram heights, indicating a substantial degree of genetic divergence from the rest of the samples. In contrast, the remaining genotypes—including various Mutant, Standard, and Parent lines—cluster together at much lower heights, suggesting that they are more closely related genetically. This pattern indicates that the majority of the population shares a core genetic background with only minor variations, while a few outlier genotypes exhibit considerable genetic differentiation. The identification of these divergent genotypes, especially those that cluster separately at high heights, may be important for further investigation, as they could possess unique alleles or traits that contribute to increased diversity within the population. Such findings can inform breeding strategies and future genetic studies by highlighting lines that may serve as valuable sources of novel variation.

**Figure 10:**
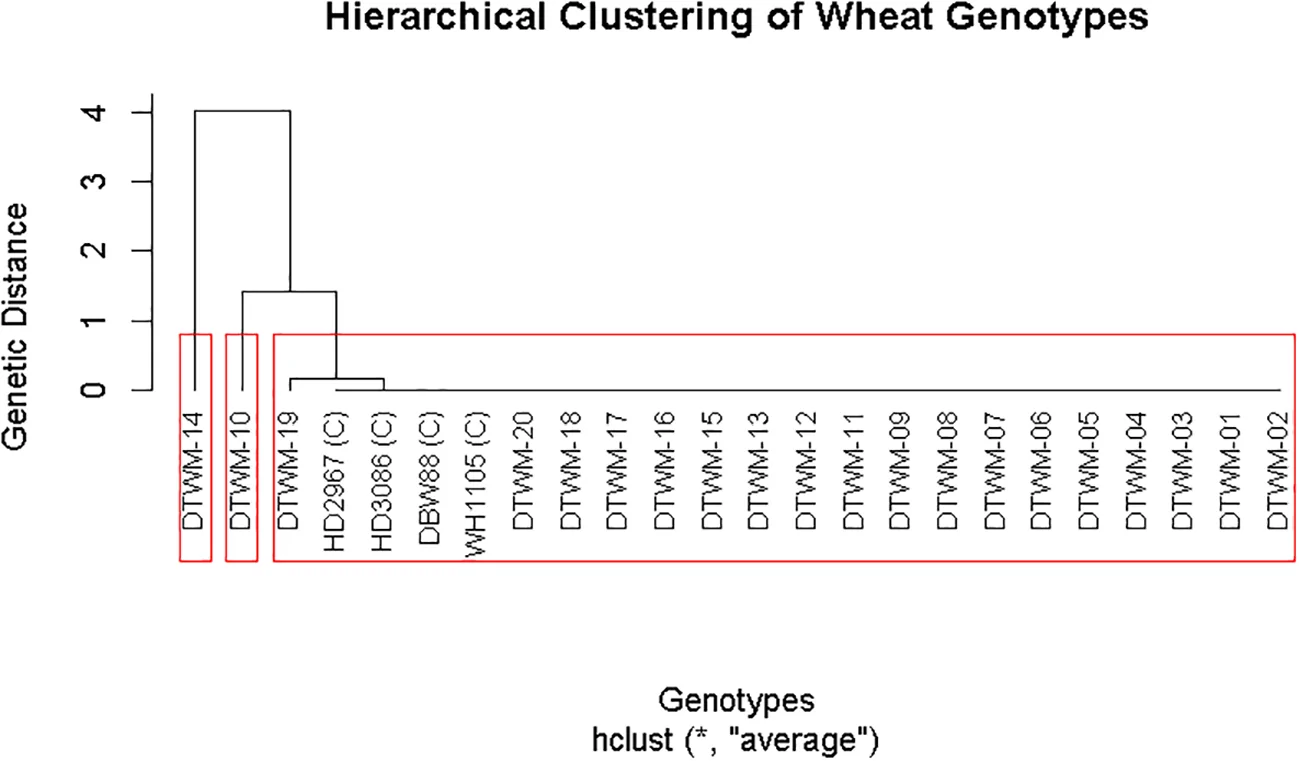
Hierarchal clustering showing genetic divergence among mutants and checks.

#### Allelic frequency distribution

snpTA00057, snpTA00051, and snpTA00027 show the highest allelic frequencies graphically (**Figure 11**), it indicates that these SNP loci have the most prevalent alleles in the population under study. High allele frequency here means the major allele at these loci is common in the genotypes analyzed, which can indicate that these SNPs are well-conserved or fixed in the population. Typically, such SNPs contribute significantly to genetic variation interpretation because their alleles are widespread, suggesting they might be important for traits under selection or neutral stability. The high frequency of particular SNP alleles implies that these loci can serve as reliable markers for population structure or diversity assessments. Moreover, it also suggests that less frequent or minor alleles at these SNP positions are less common and more unique genotypes, making these SNPs potentially useful for identifying major lineage patterns or breeding decisions. Overall, the observation of highest frequencies in snpTA00057, snpTA00051, and snpTA00027 highlights their importance as major allelic variants in population analysis. This interpretation assumes that the graphical representation reflects allele frequency distribution and that these three SNPs represent critical polymorphic loci contributing to genetic makeup and diversity within your study samples.

**Figure 11:**
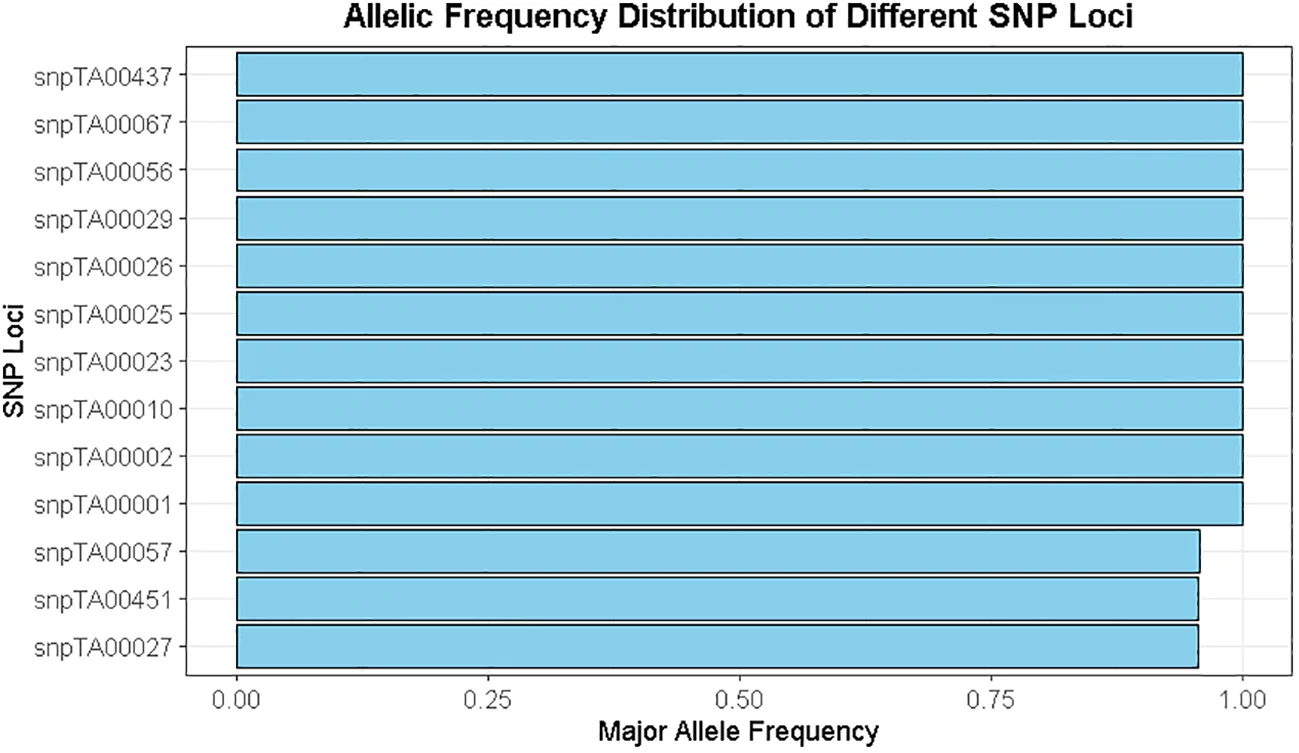
Allelic frequency distribution of different SNP loci.

### Genotype heatmap

The genotype heatmap (**Figure 12**), generated using SNP genotype calls (0 = AA, 1 = AB, 2 = BB), visualized allele distribution across samples. Predominant blue coloration (AA) indicated high homozygosity within most genotypes, typical of resistant lines. Distinct patches of green (AB) and red (BB) alleles observed in DTWM-10 and DTWM-14, pointed to heterozygosity or alternative allelic forms possibly linked with susceptibility or variable responses to yellow rust. This pattern corroborates the PCA and clustering outputs, confirming the presence of unique SNP variations among certain mutants.

**Figure 12:**
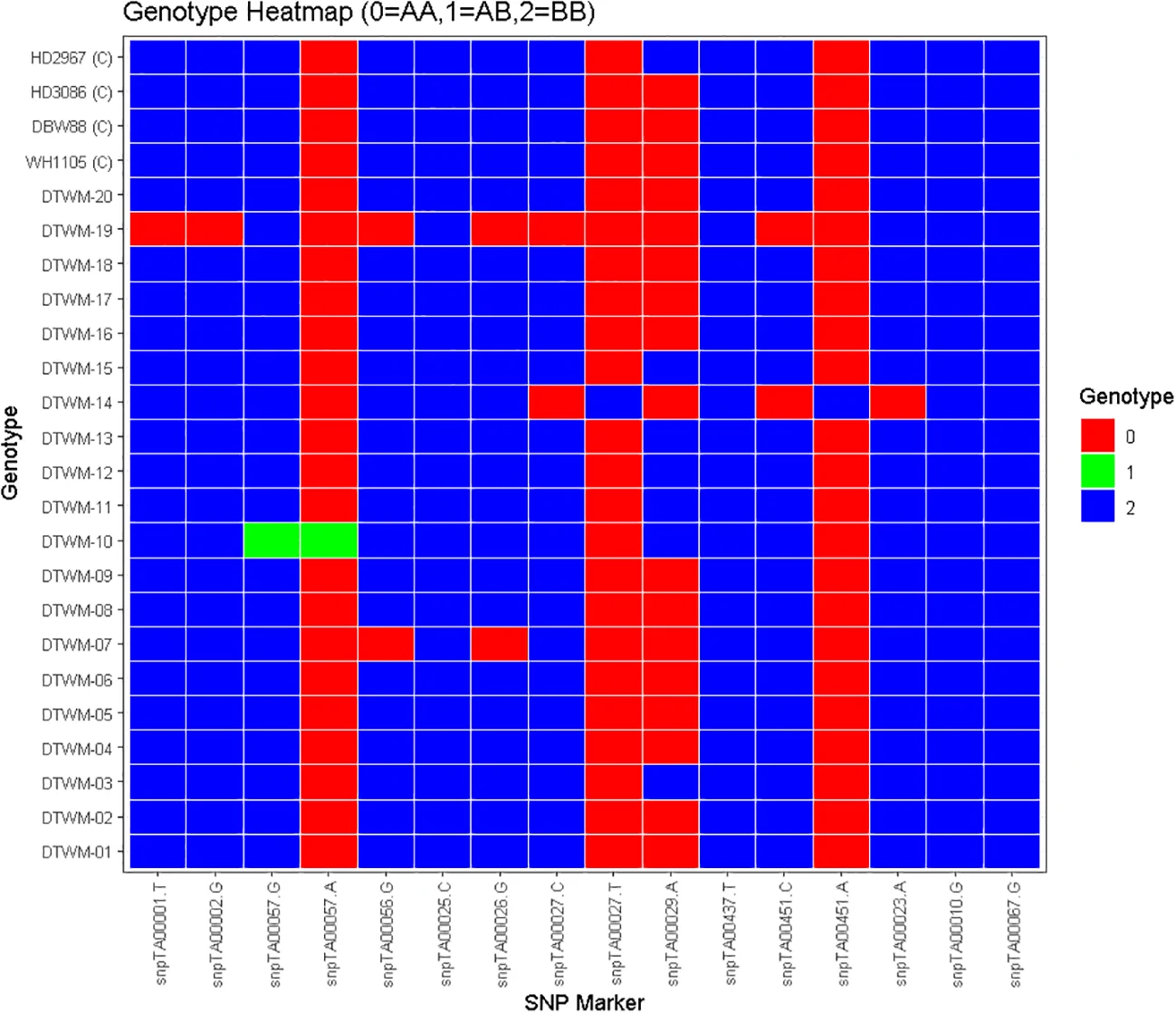
Genotype heatmap showing SNP markers across set of individuals.

The molecular analysis revealed that most mutant lines remained genetically similar to DBW88 and the other checks at the loci surveyed, whereas DTWM-10 and DTWM-14 showed comparatively greater divergence. Their separation was consistently observed in PCA, hierarchical clustering and IBS analyses, indicating distinct allele combinations within the targeted SNP marker set. These lines may therefore provide useful contrasting materials for further genetic characterization. However, because the analysis was based on a limited panel of functional SNP markers, the observed patterns should not be interpreted as a genome-wide estimate of genetic diversity or population structure.

## Discussion

### Variability and trait interrelationships

The significant differences observed in Univariate ANOVA across three crop seasons for different traits including yield and rust resistance, confirm that induced mutagenesis successfully generated novel phenotypic diversity from the parental background. Pearson correlation analysis provided pivotal insights into the physiological and morphological trade-offs regulating grain yield.

#### Yield component drivers

The highly significant positive association of grain yield with grain number per spike (GNPS) (0.55***) and grain weight (GW) (0.45***)) confirms that sink strength and overall spike productivity are the primary drivers of yield performance in the mutant lines. This strong correlation aligns with established wheat ideotype breeding paradigms, which demonstrate that maximizing grain allocation per spike is the most reliable path to genetic yield gains in semi-dwarf bread wheat. Conversely, grain yield exhibited a severe negative correlation with days to heading (DTH) (-0.54***). The negative association between days to heading and grain yield suggests that earlier heading was associated with higher yield under the conditions evaluated.

#### Spike architecture trade-offs

Interestingly, grain yield showed a high negative correlation with number of spikes (NS) (-0.55***) alongside a moderate positive correlation with spike length (SL) (0.27***). This indicates that merely increasing tiller or spike count induces aggressive intra-plant competition for resources, which ultimately reduces final grain size and weight. Breeding programs should therefore prioritize spike efficiency (higher grain density and weight per spike) over vegetative prolificacy (Kumar et al., 2019a).

### Genotype-by-environment interaction and yield stability

Yield is a complex polygenic trait that is highly vulnerable to seasonal and spatial environmental fluctuations. The AMMI ANOVA confirmed that grain yield was significantly affected (p < 0.01) by the main effects of environment (E), genotype (G) and G×E interactions. The fact that the first Interaction Principal Component Axis (IPCA1) captured more than 80% of the G×E interaction variance proves that a single-dimensional interaction axis is highly robust for explaining the adaptive patterns of these genotypes. This aspect has been highlighted by many multi-environment wheat studies, such as those by Yan and Tinker (2006); Ram et al. (2020) and Kumar et al. (2021).

To categorize these responses, the Lin and Binns (1988) superiority index ((P_{i}) was cross-referenced with AMMI and GGE biplots. It helps in identification of winning genotypes within environments in multi environment trials. The ‘which-won-where’ analysis, which graphically represents crossover GE interaction, mega-environment differentiation, and particular genotype adaptation, is the most alluring aspect of GGE biplots (Mishra et al., 2015; Oral et al., 2018). To investigate the potential existence of many mega-environments in a region, it is crucial to visualize a “which-won-where” pattern in multi-environment trials (Yan and Tinker, 2006; Gupta et al., 2023).

### Broadly adaptable genotypes

Mutant DTWM-16 recorded the highest baseline superiority across environments (P_i = 0.14), followed closely by the check variety WH1105 (P_i = 0.19) and another mutant DTWM-15 (P_i = 0.35). These findings match the AMMI2 and GGE models, which classified DTWM-16, DTWM-15 and WH1105 as stable and high yielding genotypes over the years. These genotypes represent highly buffered candidates ideal for sustainable production across unpredictable environments.

### Specific adaptation targets

Environmental partitioning using (P_{i(f)}) and (P_{i(u)}) successfully revealed niche adaptations. Genotypes WH1105 and DBW88 proved superior under favorable conditions, with AMMI1 specifically linking their high yields to the optimized environments. Conversely, mutants DTWM-08 and DTWM-16 showed outstanding adaptation under stress conditions, confirming their resilience under adverse field conditions.

### Unstable and poor performers

Mutants DTWM-12 (P_i = 2.28) and DTWM-20 (P_i = 2.07) showed massive deviations from the highest yielder genotypes. Their high IPCA1 scores and long environmental vectors confirm extreme instability or strict restriction to low-yielding environments.

The GGE “Which-Won-Where” polygon view visually validates the crossover G×E interactions. By identifying DTWM-08 and DTWM-16 as the winning genotypes in first year (E1), and WH1105 and DTWM-09 as the winners in third year (E3), the analysis confirms that the target testing seasons contained distinct testing environments. According to environmental vector analysis, E3 represents the ideal test environment due to its moderate discriminativeness and high representativeness, whereas E1 serves as an aggressive, highly discriminating environment suitable for screening strict stress-tolerant lines. This application of GGE biplots to delineate environments aligns perfectly with the landmark methodology of Yan et al. (2007), which states that an ideal test site must simultaneously differentiate genotypes while representing the average target environment.

### Yellow rust dynamics and impact on yield attributes

Stripe (yellow) rust, is a major wheat disease which poses a constant threat to wheat production not only in India but also worldwide. In this study, the presence of a severe, uniform rust infection across seasons provided an excellent baseline for adult plant response screening. The mutants displayed an exceptionally wide phenotypic spectrum, ranging from complete immunity (34% of genotypes, including DTWM-03 and DTWM-08) to high susceptibility (42% of lines, including the parent DBW88). This wide divergence demonstrates that mutation induction is an effective tool to break tight genetic linkages and generate novel resistance alleles from a susceptible parental background.

The Pearson correlation matrix evaluating the Average Coefficient of Infection (ACI) against yield components clarified the damage caused by the pathogenic infection. ACI was negatively correlated with all evaluated yield-related traits, including grain weight (GW), thousand grain weight (TGW), and total number of grains (TNG). The exceptionally strong negative correlation between ACI and TGW highlights that stripe rust damages wheat primarily by disrupting post-anthesis grain filling. Conversely, the positive correlation clusters among GW, TGW, and TNG confirm that maximizing kernel weight directly boosts overall yield. Therefore, selecting for low ACI (high rust resistance) is an absolute prerequisite to secure high grain weight and preserve yield potential in rust-prone environments. This finding is highly consistent with the physiological research of Islam et al. (2023), which demonstrates that severe *Puccinia* infections destroy green leaf area, disrupt host chlorophyll fluorescence, and accelerate leaf senescence. This drastically reduces the source-to-sink translocated photo-assimilates, directly resulting in shrivelled grains, reduced kernel hardness, and minimized thousand-grain weight.

### Molecular diversity and genomic basis of mutagenesis

Characterizing the genotypes at the molecular level using KASP (Kompetitive Allele-Specific PCR) SNP markers provided a precise genomic context to our phenotypic observations. Principal Component Analysis (PCA) based on these markers showed that PC1 (28%) and PC2 (22%) together captured 50% of the total molecular variance. The tight clustering of the majority of mutant lines around the origin, close to the parental line (DBW88) and standard checks, indicates a narrow overall genetic base. This pattern is highly characteristic of induced mutagenesis. As noted by functional genomics reviews on hexaploid wheat polyploidy, chemical mutagens like ethyl methanesulfonate (EMS) induce highly localized point mutations (typically transitions) across the subgenomes without disrupting the elite, well-conserved genetic background of the parent plant. This explains why the mutant lines retain close structural proximity to the parent while exhibiting distinct phenotypic improvements in traits like rust resistance and yield components.

However, two mutants, i.e., DTWM-10 and DTWM-14 emerged as distinct outliers, positioned far from the central cluster along both the PC1-PC2 and PC1-PC3 biplots. This clear separation was independently verified by the hierarchical clustering dendrogram, where these two lines branched out at significantly higher node heights, as well as the Identity By State (IBS) heatmap, which revealed elevated genetic distances (0.20–0.25) compared to the rest of the population (0.00–0.10).

The underlying molecular architecture of these divergent lines was clearly illustrated by the single-nucleotide polymorphism (SNP) genotype heatmap. While the stable, resistant lines exhibited uniform blocks of homozygosity (blue), mutants DTWM-10 and DTWM-14 displayed unique, contrasting patches of heterozygosity (green) and alternative homozygosity (red). These distinct molecular profiles match their variable responses to yellow rust and altered yield dynamics. Furthermore, polymorphic markers like snpTA00057, snpTA00051 and snpTA00027 showed exceptionally high allele frequencies, proving they are well-conserved loci within this studied genotype set. These specific SNPs serve as excellent high-throughput diagnostic tools for marker-assisted selection (MAS) (Kamboj et al., 2020) and pedigree tracking in wheat breeding programs.

## Conclusions

By combining multi-environmental field trials with high-throughput KASP SNP profiling, this study successfully identified elite wheat mutants that balance high yield stability with robust disease resistance. Genotypes DTWM-08, DTWM-16 and WH1105 stand out as the premier candidates for regional multi-environment utilization due to their exceptionally low (P_{i}) values, high mean yields, and stable AMMI/GGE expressions. At the same time, the highly divergent mutant lines DTWM-10 and DTWM-14 provide a valuable source of novel genetic variation. These outliers should be integrated into future hybridization panels to expand the narrow genetic base of current elite lines, break unfavorable linkage drags, and recombine novel alleles to achieve durable, long-term resistance against stripe rust.

## Data Availability

All relevant data is contained within the article. The original contributions presented in the study are included in the article, further inquiries can be directed to the corresponding author.
